# AmgRS-mediated envelope stress-inducible expression of the *mexXY* multidrug efflux operon of *Pseudomonas aeruginosa*

**DOI:** 10.1002/mbo3.226

**Published:** 2014-12-01

**Authors:** Calvin Ho-Fung Lau, Thomas Krahn, Christie Gilmour, Erin Mullen, Keith Poole

**Affiliations:** 1 Department of Biomedical and Molecular Sciences, Botterell Hall, Queen's UniversityKingston, Ontario, K7L 3N6, Canada

**Keywords:** Efflux, envelope stress, multidrug, *Pseudomonas aeruginosa*, two-component system

## Abstract

AmgRS is an envelope stress-responsive two-component system and aminoglycoside resistance determinant in *Pseudomonas aeruginosa* that is proposed to protect cells from membrane damage caused by aminoglycoside-generated mistranslated polypeptides. Consistent with this, a Δ*amgR* strain showed increased aminoglycoside-promoted membrane damage, damage that was largely absent in AmgRS-activated *amgS-*mutant strains. Intriguingly, one such mutation, V121G, while providing for enhanced resistance to aminoglycosides, rendered *P. aeruginosa* susceptible to several ribosome-targeting nonaminoglycoside antimicrobials that are inducers and presumed substrates of the MexXY-OprM multidrug efflux system. Surprisingly, the *amgS*_V__121G_ mutation increased *mexXY* expression threefold, suggesting that export of these nonaminoglycosides was compromised in the *amgS*_V__121G_ mutant. Nonetheless, a link was established between AmgRS activation and *mexXY* expression and this was confirmed in studies showing that aminoglycoside-promoted *mexXY* expression is dependent on AmgRS. While nonaminoglycosides also induced *mexXY* expression, this was not AmgRS-dependent, consistent with these agents not generating mistranslated polypeptides and not activating AmgRS. The aminoglycoside inducibility of *mexXY* was abrogated in a mutant lacking the AmgRS target genes *htpX* and PA5528, encoding a presumed cytoplasmic membrane-associated protease and a membrane protein of unknown function, respectively. Thus, aminoglycoside induction of *mexXY* is a response to membrane damage and activation of the AmgRS two-component system.

## Introduction

*Pseudomonas aeruginosa* is a common nosocomial pathogen (Hidron et al. 2008; Zhanel et al. 2008, 2010) and a major cause of morbidity and mortality in patients with cystic fibrosis (CF) (Govan et al. 2007; de Vrankrijker et al. 2010; Brugha and Davies 2011). Treatment of *P. aeruginosa* infections is complicated by the microorganism's innate resistance to many antimicrobials, a product of its impressive intrinsic resistome (Olivares et al. 2013), and its access to an array of acquired resistance mechanisms (Breidenstein et al. 2011; Poole 2011). Major contributors to antimicrobial resistance in this organism are multidrug efflux systems of the resistance-nodulation-division (RND) family, including MexAB-OprM, MexCD-OprJ, MexEF-OprN, and MexXY-OprM, which contribute to both intrinsic (MexAB-OprM, MexXY-OprM) and acquired (all) resistance (Poole 2013). MexXY-OprM is somewhat unique in *P. aeruginosa* in conferring resistance to the aminoglycoside (AG) class of antimicrobials (Sobel et al. 2003; Poole 2005a; Henrichfreise et al. 2007), a class long-used in the management of CF lung infections caused by this microorganism (Prayle and Smyth 2010). While several endogenous AG resistance determinants are present in *P. aeruginosa* (Schurek et al. 2008; Dötsch et al. 2009; Lee et al. 2009; Krahn et al. 2012), MexXY-OprM is the predominant mechanism of resistance to these agents in CF isolates (Poole 2005a; Henrichfreise et al. 2007; Vettoretti et al. 2009).

The MexXY-OprM efflux system is comprised of a cytoplasmic membrane (CM) drug-proton antiporter (MexY), an outer membrane porin (OprM) and a periplasmic membrane fusion protein that joins the membrane-associated components together (MexX) (Aires et al. 1999). The MexX and MexY components are encoded by a single operon under the control of an adjacent repressor gene, *mexZ* (Aires et al. 1999; Matsuo et al. 2004), while OprM, which functions as the outer membrane component of several multidrug efflux systems in *P. aeruginosa* (Poole 2005b), is encoded by the 3rd gene of another multidrug efflux operon, *mexAB-oprM* (Aires et al. 1999; Mine et al. 1999). The *mexXY* operon is antimicrobial inducible, with only those agents known to target the ribosome able to promote *mexXY* expression (Masuda et al. 2000a; Jeannot et al. 2005; Morita et al. 2006). Antimicrobial-inducible *mexXY* expression is compromised by so-called ribosome protection mechanisms (Jeannot et al. 2005), suggesting that the MexXY efflux system is recruited in response to ribosome disruption or defects in translation. Consistent with this, mutations in *fmt* (encoding a methionyl-tRNA-formyltransferase) (Caughlan et al. 2009), *folD* (involved in folate biosynthesis and production of the formyl group added to initiator methionine) (Caughlan et al. 2009), and the ribosomal protein genes *rplA* (Westbrock-Wadman et al. 1999), *rplY* (El'Garch et al. 2007), and the *rplU*-*rpmA* operon (Lau et al. 2012), all of which are expected to negatively impact protein synthesis, increase the expression of *mexXY*. Upregulation of *mexXY* by antimicrobials (Morita et al. 2006) or mutations (*fmt*/*folD* [Caughlan et al. 2009], *rplY* [El'Garch et al. 2007] and *rplU*-*rpmA* [Lau et al. 2012]) is dependent upon a gene, *armZ* (formerly known as PA5471), encoding a MexZ-targeting anti-repressor (Yamamoto et al. 2009; Hay et al. 2013). Expression of *armZ* is also promoted by ribosome-disrupting antimicrobials (Morita et al. 2006) and *fmt*/*folD* (Caughlan et al. 2009) or *rplU*-*rpmA* (Lau et al. 2012) mutations. Moreover, *armZ* expression is governed by a transcriptional attenuation mechanism that directly links ribosome/translation disruption and *armZ* expression, providing a mechanism whereby ribosome perturbation drives MexXY recruitment (Morita et al. 2009). Still, drug-inducible *mexXY* expression independent of MexZ (Hay et al. 2013) and ArmZ (Muller et al. 2010) has been reported, an indication that additional regulator(s) influence *mexXY* expression. Indeed, the ParRS two-component system (TCS) implicated in adaptive resistance to cationic antimicrobial peptides, such as the polymyxins (Fernandez et al. 2010), has been linked to ArmZ-independent *mexXY* expression (Muller et al. 2010), with mutations in the *parRS* locus driving *mexXY* expression and AG resistance (Muller et al. 2010; Guenard et al. 2014).

Although ArmZ is required for *mexXY* induction in response to ribosome perturbation, it is insufficient for maximal drug-inducible expression of this efflux operon – *armZ*/PA5471 hyperexpression from a multicopy plasmid or resultant from a mutation in the *armZ* leader peptide both provide for much more modest *mexXY* expression as compared with drug-treated cells (Morita et al. 2006). Presumably, additional downstream effects of ribosome perturbation work in concert with ArmZ to effect/promote *mexXY* derepression. In the case of AGs, which promote mistranslation (Weisblum and Davies 1968), this may relate to the generation of aberrant polypeptides that damage the CM (Davis et al. 1986; Busse et al. 1992). Interestingly, the AmgRS TCS (Lee et al. 2009) in *P. aeruginosa* that appears to be operationally similar to the CpxRA envelope stress response TCS in *Escherichia coli* (Ruiz and Silhavy 2005) has been proposed to control an adaptive response to membrane damage caused by AG-generated aberrant polypeptides (Lee et al. 2009). Contributing to intrinsic AG resistance (Lee et al. 2009) this TCS has also been linked to acquired resistance in both laboratories and clinical AG-resistant strains as a result of gain-of-function “activating” mutations in the *amgS* gene that encodes the sensor kinase component of this TCS (Lau et al. 2013). In the current study we confirm that AmgRS, indeed, provides protection against AG-promoted membrane damage. We further demonstrate that AmgRS activation via an *amgS* gain-of-function mutation or AG exposure promotes *mexXY* expression, and that AmgRS mediates *mexXY* expression in response to AGs, but not to other inducers of this efflux operon.

## Experimental Procedures

### Bacterial strains and growth conditions

The bacterial strains and plasmids used in this study are described in Table1. Bacterial cells were cultured in Luria broth (L-broth) and on Luria agar (L-agar), with antibiotics as necessary, at 37°C. In *E. coli*, plasmid pEX18Tc and its derivatives were maintained or selected with 10 *μ*g/mL tetracycline, while plasmid pK18mobsacB and its derivatives were maintained or selected with 50 *μ*g/mL kanamycin.

**Table 1:** Bacterial strains and plasmids used in this study.

Strain or plasmid	Description^1^	Reference
*Escherichia coli* strains		
DH5*α*	φ80d *lacZ*ΔM15 *endA1 recA1 hsdR17* (r_K_^−^ m_K_^+^) *supE44 thi- 1 gyrA96 relA1* F^−^Δ(*lacZYA-argF*) *U169*	Ausubel et al. (1992)
S17-1	*thi pro hsdR recA* Tra^+^	Simon et al. (1983)
*Pseudomonas aeruginosa* strains		
K767	PAO1 prototroph (wild-type)	Masuda and Ohya (1992)
K3249	K767 derivative carrying the *amgS*_R182C_ mutation	Lau et al. (2013)
K3260	K767 derivative carrying the *amgS*_V121G_ mutation	Lau et al. (2013)
K3159	K767Δ*amgR*	Krahn et al. (2012)
K3583	K767Δ*amgS*	This study
K3584	K3249Δ*amgR*	This study
K3585	K3260Δ*amgR*	This study
K1525	K767Δ*mexXY*	De Kievit et al. (2001)
K3586	K1525 derivative carrying the *amgS*_V121G_ mutation	This study
K2413	K767Δ*armZ*	Morita et al. (2006)
K3587	K2413 derivative carrying the *amgS*_V121G_ mutation	This study
K2415	K767Δ*mexZ*	Morita et al. (2006)
K3615	K2415Δ*amgR*	This study
K3589	K767Δ*yccA*	This study
K3590	K767Δ*htpX*	This study
K3591	K767ΔPA5528	This study
K3593	K767Δ*htpX*Δ*yccA*	This study
K3594	K767 Δ*htpX*ΔPA5528	This study
K3595	K767 Δ*yccA*ΔPA5528	This study
K3596	K767Δ*htpX*ΔPA5528Δ*yccA*	This study
Plasmids		
pEX18Tc	Broad-host-range gene replacement vector; *sacB* Tc^r^	Hoang et al. (1998)
pK18mobsacB	Broad-host-range gene replacement vector; *sacB* Kan^r^	Schäfer et al. (1994)
pCL6	pEX18Tc derivative carrying *amgS*_V121G_	Lau et al. (2013)
pCG005	pEX18Tc::Δ*amgR*	Krahn et al. (2012)
pCL22	pK18mobsacB::Δ*amgR*	This study
pCL23	pEX18Tc::Δ*amgS*	This study
pCL24	pEX18Tc::Δ*yccA*	This study
pCL25	pEX18Tc::Δ*htpX*	This study
pCL26	pEX18Tc::ΔPA5528	This study

1 Tc^r^, tetracycline-resistant; Kan^r^, kanamycin resistant.

### DNA methods

Standard protocols were used for restriction endonuclease digestion, ligation, transformation, and agarose gel electrophoresis, as described by Sambrook and Russell (Sambrook and Russell 2001). Plasmid and chromosomal DNA was prepared as before (Lau et al. 2012). DNA fragments used for cloning were extracted from agarose gels using a Wizard® SV gel and PCR clean-up system (Fisher Scientific, Ltd., Nepean, Canada). CaCl_2_-competent *E. coli* cells were prepared as described previously (Inoue et al. 1990). Oligonucleotide synthesis was carried out by Integrated DNA Technologies (Coralville, IA). Nucleotide sequencing was carried out by ACGT Corp. (Toronto, Canada) using universal primers.

### Construction of *P. aeruginosa* gene-deletion mutants

*Pseudomonas aeruginosa* strains carrying various gene deletions were generated by first engineering the deletions in plasmid pEX18Tc (or plasmid pK18mobsacB in the case of the Δ*amgR* strain K3585) and then mobilizing them into *P. aeruginosa* from *E. coli* S17-1 as before (Srikumar et al. 1997; Krahn et al. 2012). Deletions were constructed by amplifying, via PCR, 1-kb fragments upstream and downstream of the sequences being deleted and cloning these individually into plasmid pEX18Tc for sequencing (to ensure that no mutations had been introduced during PCR) and then together into pEX18Tc to generate the deletion construct. PCR fragments were gel purified and digested with restriction enzymes (sites incorporated into the PCR primers) prior to cloning into appropriately digested plasmids. For deletions in *amgS*, *htpX*, *yccA,* and PA5528 the upstream and downstream fragments were amplified using the corresponding UP-F and UP-R, and DN-F and DN-R primers, respectively, (Table2) in separate 50-*μ*L reaction mixtures containing 10 ng of *P. aeruginosa* K767 chromosomal DNA, 1 U of Phusion high-fidelity DNA polymerase (New England BioLabs, Ltd., Pickering, Ontario, Canada), 1X Phusion HF buffer, 5% (vol/vol) dimethyl sulfoxide (DMSO), primers at a 0.6 *μ*mol/L final concentration, and deoxynucleoside triphosphates (dNTPs) at a 0.2 mmol/L final concentration. The mixtures were heated for 3 min at 98°C, followed by 35 cycles of 0.5 min at 98°C, 0.5 min at 72°C, and 0.5 min at 72°C, before finishing with 10 min at 72°C. For Δ*amgR*, the previously constructed *amgR* deletion plasmid pCG005 was employed as described (Krahn et al. 2012) or the Δ*amgR* gene was excised from pCG005 as a single 2-kb EcoRI-PstI restriction fragment and cloned into plasmid pK18mobsacB to generate an alternat-ive Δ*amgR* vector. *Pseudomonas aeruginosa* transconjugants harboring chromosomal inserts of the pEX18Tc- or pK18mobsacB-derived deletion vectors were selected on L-agar plates containing tetracycline (50 *μ*g/mL) and chloramphenicol (5 *μ*g/mL; to counterselect *E. coli* S17-1), or kanamycin (1500 *μ*g/mL) and chloramphenicol (10 *μ*g/mL; counterselect *E. coli* S17-1), respectively. These were subsequently streaked onto L-agar containing sucrose (10% [wt/vol]), with sucrose-resistant colonies screened for the appropriate deletion using colony PCR with 2.5 U Taq polymerase in 10% (vol/vol) DMSO (Sheu et al. 2000). Colony PCR was carried out using either the respective UP-F and DN-R primers (Δ*amgR*, Δ*amgS,* and ΔPA5528; Table2), or the scr-F and scr-R primer set (Δ*yccA* and Δ*htpX*; Table2) for each deletion. The reaction mixtures were heated for 3 min at 95°C, followed by 30 cycles of 30 sec at 95°C, 30 sec at either 66.8°C (for Δ*amgS*), 65°C (for Δ*amgR*), 60°C (for Δ*yccA*), 55°C (for Δ*htpX*) or 62°C (for ΔPA5528), and either 3.5 min (for Δ*amgS*), 3 min (for Δ*amgR* and ΔPA5528) or 2 min (for Δ*yccA* and Δ*htpX*) at 72°C, before finishing with 10 min at 75°C.

**Table 2:** Oligonucleotides used in this study.

Primer	Oligonucleotide sequence (5′→3′)^1^	Reference
amgRUp-F	GACTGAATTCCTGTAGAAGTCCTGGCGGT	Krahn et al. (2012)
amgRDown-R	GACTCTGCAGCGGCGCTGGAGAAACTGGT	Krahn et al. (2012)
amgSUP-F	GGACGTGAATTCGACTTGCTGCGGTTGAAC	This study
amgSUP-R	GACCTGTCTAGAACCAGAGCGGCGTTTTC	This study
amgSDN-F	GGCAGTTCTAGATGATACCCGACGGGTTTG	This study
amgSDN-R	AACGCTAAGCTTGACAGAAGGTCCATGCCAC	This study
yccAUP-F	GCAGTTGAGCTCTGGTACTGGTGGACGACGATG	This study
yccAUP-R	GCAGTTGGATCCTTGCATGGTGTGGGTACTCC	This study
yccADN-F	GAGGCTGGATCCGGCGACGACTGATAGAAAAC	This study
yccADN-R	GAGGCATCTAGAGGGTTGAGCAGGTACAGGCA	This study
yccA-scr-F	CCGTTGCCTTAAACCACTCG	This study
yccA-scr-R	AATCACCTGGTCGTAGCGTTG	This study
htpXUP-F	CATCGTGAGCTCGAAGGTCTCGACATCCTCG	This study
htpXUP-R	CATCGTGGTACCTAATTCTGGCCGGTGAAG	This study
htpXDN-F	CATCGTGGTACCTCCGCTTTCACACTTGGGA	This study
htpXDN-R	CGTGAGTCTAGAACGCCAGGCAGTCGTAG	This study
htpX-scr-F	CGCCAACACCATTCATTACG	This study
htpX-scr-R	TGAACCATTCGGCCATGTG	This study
PA5528UP-F	CATCGTGAGCTCGAACTTCGCCACGTAGG	This study
PA5528UP-R	GCACTCGGTACCAGAACACGCTGCATTGTAG	This study
PA5528DN-F	CATCGTGGTACCGAACTGATCCCGAGTGGTTG	This study
PA5528DN-R	GGAGCTTCTAGATGGTGTCGCCATTCAGC	This study
qPCR-htpX-F	ATCTCCAAGTGGATGGCGA	Lau et al. (2013)
qPCR-htpX-R	CAGCTCTTCGACGGTTTGC	Lau et al. (2013)
qPCR-PA5528-F	ATGCAGCGTGTTCTCAGC	Lau et al. (2013)
qPCR-PA5528-R	CGCTTGGCATTGGCATCCA	Lau et al. (2013)
qPCR-mexX-F	CTATCGGCATCACCAGCG	Lau et al. (2012)
qPCR-mexX-R	ATCTGGAACAGCACGGTG	Lau et al. (2012)
qPCR-PA5471-F	CATCAAGCCTTTGTCCGC	Lau et al. (2012)
qPCR-PA5471-R	CGGTGGTTTGCAGTTGCT	Lau et al. (2012)
qPCR-rpoD-F	ATCCTGCGCAACCAGCAGAA	Lau et al. (2012)
qPCR-rpoD-R	TCGACATCGCGCGGTTGATT	Lau et al. (2012)

1 Restriction endonuclease cleavage sites are underlined.

### Construction of *P. aeruginosa* AmgS_V121G_ mutants

To introduce the *amgS*_V121G_ mutation into the *P. aeruginosa* Δ*mexXY* and Δ*armZ* strains K1525 and K2413, respectively, plasmid pCL6, a pEX18Tc-based gene replacement vector carrying the *amgS*_V121G_ mutation, was mobilized into the *P. aeruginosa* deletion strains from *E. coli* S17-1 and *amgS*_V121G_ derivatives selected as before (Lau et al. 2013).

### Membrane depolarization assay

A previously described fluorometric assay (Krahn et al. 2012), involving the membrane potential-sensitive dye bis-(1,3-dibutylbarbituric acid) trimethine oxonol [DiBAC_4_(3)], was employed to measure the degree of CM depolarization promoted by AG treatment of *P. aeruginosa*. Briefly, early logarithmic phase (optical density at 600 nm [OD_600 nm_] = 0.3–0.5) L-broth subcultures of *P. aeurginosa* were treated with the AGs gentamicin (2 or 5 *μ*g/mL final concentration) or tobramycin (0.5 or 1.25 *μ*g/mL final concentration) or the aminocyclitol, spectinomycin (SPC) (1280 *μ*g/mL). In some experiments *P. aeruginosa* was pretreated with chloramphenicol (128 *μ*g/mL) for 15 min prior to the addition of gentamicin. Samples (5 mL) of the AG-treated and untreated control cultures were taken immediately and then hourly over 3 h and exposed to DiBAC_4_(3) (Invitrogen, Burlington, Ontario, Canada) at 37°C for 5 min in the dark at a final concentration of 10 *μ*g/mL. Bacteria were then pelleted and resuspended in phosphate-buffered saline (Nehme et al. 2004) to a final OD_600 nm_ of 0.1. Membrane depolarization-dependent fluorescence emitted by cells was then measured using a Varian (now Agilent, Mississauga, Ontario, Canada) Cary Eclipse fluorescent spectrophotometer with excitation and emission wavelengths of 490 and 518, respectively.

### Quantitative real-time PCR

Bacterial RNA was isolated, purified, and reverse transcribed into cDNA as described previously (Lau et al. 2012). The primers used in quantitative real-time PCR (qPCR designation, Table2) were designed to amplify specific gene fragments with lengths of 99 bp (*htpX*), 74 bp (PA5528), 142 bp (*mexX*), 89 bp (*armZ/*PA5471), or 91 bp (*rpoD*), and were validated and described previously (Lau et al. 2012, 2013). All quantitative real-time PCR primer sets used in the present study had a minimum 4-log_10_ dynamic range. The expression of *htpX,* PA5528, *mexX, armZ,* and *rpoD* was assessed by quantitative real-time PCR as described previously using a CFX96 real-time PCR detection system (Bio-Rad, Mississauga, Ontario, Canada) (Lau et al. 2012). For each gene studied, at least one control reaction with no cDNA template was included in each experiment to check for contamination of reagent(s) and to identify unintended amplification products (e.g., primer dimers). The levels of expression of the target genes in each strain studied, normalized against that of the reference gene, *rpoD*, were calculated using the standard analysis feature of the CFX-manager software version 3.0 (Bio-Rad) and were reported herein as fold change relative to that in the *P. aeruginosa* PAO1 wild-type (WT) strain K767, unless otherwise specified.

### Antibiotic susceptibility assay

The susceptibility of *P. aeruginosa* to antimicrobial agents was assessed using the twofold serial microtiter broth dilution method described previously (Jo et al. 2003), with an inoculum of ∼5 × 10^5^ cells per mL. MICs were recorded as the lowest concentration of antibiotic inhibiting visible growth after 18 h of incubation at 37°C.

## Results

### AmgRS is a membrane damage-responsive TCS

**Figure 1:**
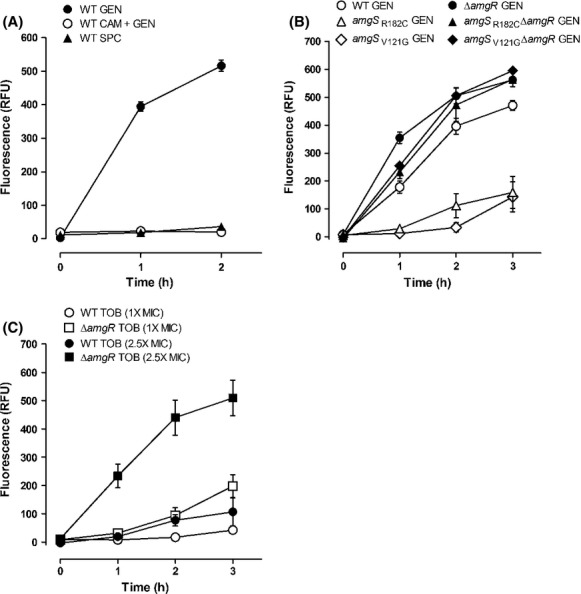
Aminoglycoside-promoted cytoplasmic membrane depolarization. Cytoplasmic membrane depolarization, as assessed by DiBAC_4_(3) fluorescence, was measured over time following exposure of *Pseudomonas aeruginosa* to various antimicrobials added at T = 0 h. (A) Wild-type (WT) *P. aeruginosa* strain K767 exposed to 5 *μ*g/mL (2.5X MIC) of gentamicin (GEN; filled circles), 128 *μ*g/mL chloramphenicol + 5 *μ*g/mL gentamicin (CAM + GEN; open circles) and 1280 *μ*g/mL (2.5X MIC) spectinomycin (SPC; filled triangles). (B) *Pseudomonas aeruginosa* strains K767 (WT; open circles), K3159 (Δ*amgR*; filled circles), K3249 (*amgS*_R__182C_; open triangles), K3584 (*amgS*_R__182C_ Δ*amgR*; filled triangles), K3260 (*amgS*_V__121G_; open diamonds), and K3585 (*amgS*_V__121G_ Δ*amgR*; filled diamonds) exposed to 2 *μ*g/mL (1X MIC) of gentamicin (GEN). (C) *Pseudomonas aeruginosa* strains K767 (WT; circles) and K3159 (Δ*amgR*; squares) exposed to 0.5 *μ*g/mL (1X MIC) (open symbols) and 1.25 *μ*g/mL (2.5X MIC) (filled symbols) tobramycin (TOB). The data are means ± standard errors of the means (SEMs) of three to four independent experiments and have been corrected for fluorescence measured in the absence of antimicrobial exposure.

A recent study showed that treatment of *P. aeruginosa* with the AG gentamicin-promoted depolarization of the CM and that this was exacerbated in a mutant lacking the AmgR component of the AmgRS TCS (Krahn et al. 2012). These data were interpreted as AG-generated mistranslated polypeptides damaging the CM, and AmgRS playing a role in ameliorating this damage. Consistent with AG-generated mistranslated/polypeptides being responsible for the observed CM depolarization, exposure of wild-type *P. aeruginosa* K767 to SPC, an aminocyclitol that is related to AGs but does not promote mistranslation (Wallace et al. 1974), failed to promote membrane depolarization (Fig.1A). Moreover, treatment of *P. aeruginosa* K767 with chloramphenicol, a translation inhibitor (Vazquez 1974), prior to the addition of gentamicin abrogated gentamicin-promoted membrane depolarization (Fig.1A), again consistent with (mis)translated polypeptides being central to the membrane damage being measured in the depolarization assay. In agreement with the previous study, loss of *amgR* enhanced membrane depolarization promoted by the AGs gentamicin (modestly at 2.5X MIC [data not shown] as seen before [Krahn et al. 2012] and much more strikingly at 1X MIC [Fig.1B, compare WT and Δ*amgR*]) and, especially, tobramycin (Fig.1C), which was markedly less membrane damaging than gentamicin at a comparable MIC (Fig.1C). In addition, *amgS* gain-of-function mutants K3249 (*amgS*_R182C_) and K3260 (*amgS*_V121G_), in which the AmgRS TCS is activated and AG resistance enhanced (Lau et al. 2013), showed reduced membrane depolarization, dependent on AmgR (Fig.1B). Taken together, these data support the conclusion that AmgRS protects the CM from damage(s) caused by AG-generated, mistranslated aberrant polypeptides.

### Impact of *amgS*_V121G_ on expression of the *mexXY* multidrug efflux operon

**Figure 2:**
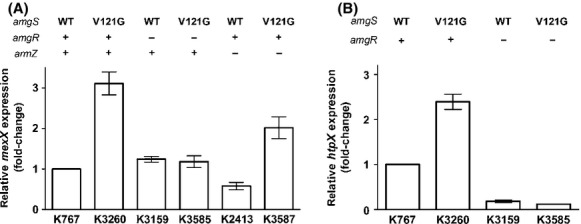
Impact of the *amgS*_V__121G_ mutation on *mexXY* and *htpX* expression. Expression of (A) *mexXY* and (B) *htpX* was assessed in late-log phase cultures of the indicated strains using real-time quantitative PCR. The status of the *amgS* (wild-type [WT] or V121G mutation) and the *amgR* and *armZ* genes (+, wild-type gene present; −, gene deleted) in each strain is indicated. Expression was normalized to *rpoD* and is reported relative to the wild-type *Pseudomonas aeruginosa* strain K767 (fold-change). Values are means ± standard errors of the means (SEMs) from at least three independent determinations, each performed in triplicate.

While the *amgS*_R182C_ and *amgS*_V121G_ mutations provided for a modest increase in resistance to AGs (Lau et al. 2013), evaluation of their impact on susceptibility to non-AGs revealed an unexpected decrease in resistance to several agents for the *amgS*_V121G_ mutant (Table3). Interestingly, those agents for which resistance was reduced (SPC, chloramphenicol, tetracycline, and erythromycin) are known inducers (Masuda et al. 2000a; Jeannot et al. 2005; Morita et al. 2006) and probable substrates (Aires et al. 1999; Mine et al. 1999; Masuda et al. 2000b) of the MexXY-OprM multidrug efflux system. This suggested that the *amgS*_V121G_ mutation was adversely affecting the induction of *mexXY* by these agents. Examination of *mexXY* expression using quantitative real-time PCR, however, revealed that *mexXY* expression was actually enhanced ca. threefold in the *amgS*_V121G_ mutant relative to its parent strain K767 (Fig.2A). Moreover, this enhanced expression was wholly dependent on AmgR (Fig.2A), an indication that the *mexXY* upregulation was a direct result of AmgRS activation by the V121G mutation present in AmgS. This precisely mirrored the AmgR-dependent increase in the expression of *htpX* (Fig.2B), an established AmgRS-regulated gene (Lau et al. 2013). Since drug-inducible *mexXY* expression is invariably dependent on the MexZ anti-repressor, ArmZ (Morita et al. 2006; Hay et al. 2013), the dependence of *amgS*_V121G_ mutational upregulation of *mexXY* on ArmZ was also assessed. Surprisingly, while loss of *armZ* had a modest negative impact on *mexXY* expression in both the WT and V121G *amgS* backgrounds relative to their ArmZ^+^ counterparts, the *amgS*_V121G_ mutation still provided for a threefold increase in *mexXY* expression in the absence of *armZ* (Fig.2A). Thus, *mexXY* expression promoted by the *amgS*_V121G_ mutation was independent of this anti-repressor. In agreement with this, and in contrast to drug-inducible *mexXY* expression where *armZ* is upregulated in parallel with *mexXY* (Morita et al. 2006), the *amgS*_V121G_ mutation had no impact on *armZ* expression (data not shown).

**Table 3:** Impact of the *amgS*_V121G_ mutation on resistance of *Pseudomonas aeruginosa* to nonaminoglycosides.

Strain	AmgS^1^	MexXY^1^	Minimal inhibitory concentration (*μ*g/mL) for:^2^				
			SPC	TET	ERY	CAM	CAR
K767	WT	+	1024	16	512	64	64
K3249	R182C	+	1024	16	512	64	64
K3260	V121G	+	256	8	256	32	64
K1525	WT	−	64	16	128	64	64
K3586	V121G	−	64	16	128	32	64

1 The status of the AmgS (wild-type [WT] or with the indicated amino acid substitution) and MexXY (+, present; −, absent) components of the indicated strains is shown.

2 SPC, spectinomycin; TET, tetracycline; ERY, erythromycin; CAM, chloramphenicol; CAR, carbenicillin.

A possible explanation for the apparent disconnect between the increased susceptibility to non-AG MexXY-OprM substrates and the increase in *mexXY* expression was that the reduced resistance of the *amgS*_V121G_ strain K3260 was, in fact, unrelated to *mexXY*. To assess this, the *mexXY* genes were deleted in strain K3260 (yielding strain K3586) and susceptibility to these non-AGs reexamined and compared with a Δ*mexXY* strain carrying a WT *amgS* gene (strain K1525). As seen in Table3, while the *mexXY*-mutant strains were both more susceptible to these non-AG *mexXY* inducers/substrates, there was no longer any difference in susceptibility to these agents between the *amgS*_WT_ and the *amgS*_V121G_ derivatives. Thus, the enhanced drug susceptibility seen in the MexXY^+^ *amgS*_V121G_ strain K3260 was dependent on the presence of *mexXY*.

Given the increased *mexXY* expression seen in strain K3260, it was possible that the enhanced AG resistance of this mutant was attributable to MexXY-OprM and not AmgRS-mediated expression of its other target genes (e.g., *htpX*, PA5528). To assess this, the impact of the *amgS*_V121G_ mutation on AG resistance in a Δ*mexXY* background was examined. As seen in Table4, the *amgS*_V121G_ mutation promoted a comparable and, in some instances (e.g., for tobramycin and streptomycin), greater increase in AG resistance in the MexXY^−^ derivative K3586 than in the MexXY^+^ derivative K3260 (although the absolute level of resistance was lower owing to the absence of the AG-accommodating multidrug efflux system in the MexXY^−^ strains). Thus, while MexXY-OprM clearly contributes to AG resistance in the *amgS*_V121G_ strain K3260, the increase in AG resistance seen in K3260 relative to its wild-type parent strain K767 is not explained by the increased *mexXY* expression in the mutant.

**Table 4:** Impact of the *amgS*_V121G_ mutation on aminoglycoside resistance of *Pseudomonas aeruginosa*.

Strain	AmgS^1^	MexXY^1^	Minimal inhibitory concentration (*μ*g/mL) for:^2^				
			TOB	GEN	STR	PAR	NEO
K767	WT	+	1	2	32	256	32
K3260	V121G	+	2	4	64	512	64
K1525	WT	−	0.5	1	2	16	8
K3586	V121G	−	2	2	8	32	16

1 The status of the AmgS (wild-type [WT] or with the indicated amino acid substitution) and MexXY (+, present; −, absent) components of the indicated strains is shown.

2 TOB, tobramycin; GEN, gentamicin; STR, streptomycin; PAR, paromomycin; NEO, neomycin.

### AG induction of *mexXY* is AmgRS-dependent

**Figure 3:**
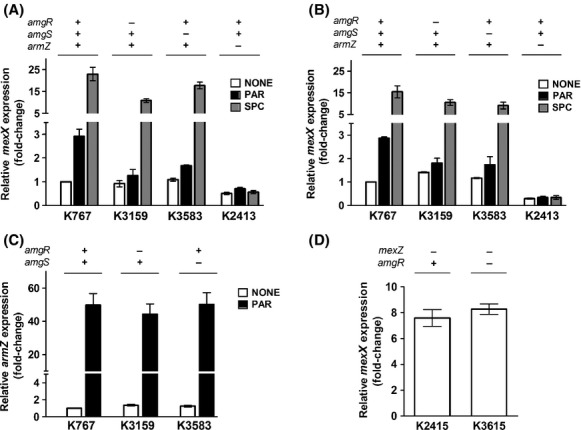
Impact of AmgRS on paromomycin-inducible *mexXY* expression. Expression of *mexXY* (A, B, and D) and *armZ* (C) was assessed in late- (A and C) and mid- (B and D) log phase cultures of the indicated strains without (open bars) or with (filled bars) exposure to paromomycin (PAR; black bars) or spectinomycin (SPC; gray bars) using real-time quantitative PCR. The status of the *amgR*, *amgS, armZ,* and *mexZ* genes (+, wild-type gene present; −, gene deleted) in each strain is indicated. Expression was normalized to *rpoD* and is reported relative to the wild-type *Pseudomonas aeruginosa* strain K767 (fold-change). Values are means ± standard errors of the means (SEMs) from at least three independent determinations, each performed in triplicate.

**Figure 4:**
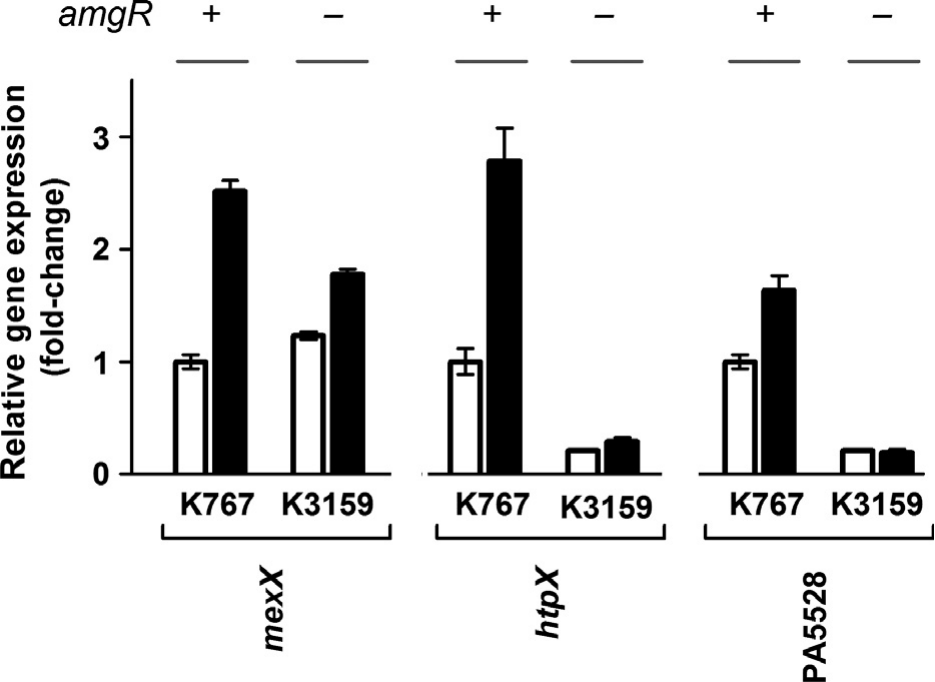
Impact of gentamicin on expression of AmgRS target genes. Expression of *mexXY*, *htpX,* and PA5528 was assessed in late-log phase cultures of the indicated strains without (open bars) or with (filled bars) exposure to gentamicin. The status of the *amgR* gene (+, wild-type gene present; −, gene deleted) in each strain is indicated. Expression was normalized to *rpoD* and is reported relative to the wild-type *Pseudomonas aeruginosa* strain K767 (fold-change). Values are means ± standard errors of the means (SEMs) from at least three independent determinations, each performed in triplicate.

Given that mutational activation of the AmgRS TCS upregulated *mexXY* expression, we questioned whether the previously reported induction of *mexXY* by AGs (Masuda et al. 2000a; Jeannot et al. 2005; Morita et al. 2006), the only agents known to activate AmgRS (Lau et al. 2013), was similarly AmgRS dependent. Initially, since the original studies of AG activation of AmgRS involved the use of late-log phase cells and 1X MIC of AGs such as paromomycin (PAR) and neomycin (Lau et al. 2013) (vs. mid-log phase cells and ¼ MIC of AGs which was used previously for inducing *mexXY* expression [Morita et al. 2006]), the potential involvement of AmgRS in AG induction of *mexXY* was assessed using late-log phase cells and 1X MIC of PAR. In agreement with previous reports of AGs inducing *mexXY* expression (Masuda et al. 2000a; Jeannot et al. 2005; Morita et al. 2006), PAR-induced *mexXY* expression in wild-type *P. aeruginosa* strain K767 (threefold; Fig.3A). PAR-inducible *mexXY* expression was, however, lacking in *amgR* and *amgS* deletion strains (Fig.3A), an indication that AG induction of *mexXY* expression was dependent on AmgRS. As expected, SPC strongly stimulated *mexXY* expression in K767 (20-fold; Fig.3A), but this was minimally impacted by the loss of *amgR* or *amgS* (Fig.3A), consistent with SPC not activating AmgRS (Lau et al. 2013) and so not stimulating *mexXY* expression via this TCS. PAR (and SPC) induction of *mexXY* was, as expected, also dependent on *armZ* – *mexXY* expression was lost in the Δ*armZ* strain K2413 (Fig.3A). Comparable results were seen when mid-log phase cells were exposed to ¼ MIC of PAR as in previous studies of AG-inducible *mexXY* expression (Fig.3B), an indication that AG-inducible *mexXY* expression is generally dependent on AmgRS, regardless of growth phase or inducing drug concentration. To confirm and extend this observation, the AmgRS dependence of AG-inducible *mexXY* expression was assessed using an additional AG, gentamicin, which was previously shown to induce *mexXY* expression (Jeannot et al. 2005). As seen in Figure4, gentamicin-induced *mexXY* expression, and this was dependent on AmgR. Moreover, and consistent with gentamicin activating this TCS, expression of the AmgRS target genes *htpX* and PA5528 was increased two- to threefold by gentamicin, and this was also dependent on AmgR (Fig.4).

AG-inducible *mexXY* expression is ultimately dependent on AG induction of *armZ* (Morita et al. 2006). Although not previously studied, we show here that PAR also induces expression of this anti-repressor (Fig.3C). Interestingly, such induction was not compromised by loss of *amgR* or *amgS* (Fig.3C), an indication that the AmgRS involvement in AG-inducible *mexXY* expression is independent of MexZ (i.e., does not promote *mexXY* expression by influencing ArmZ-modulation of MexZ repressor activity). Consistent with MexZ and AmgRS regulating *mexXY* expression independently and AmgRS being required only for AG-promoted *mexXY* expression, the elevated, drug-independent expression of *mexXY* seen in the Δ*mexZ* strain K2415 was not lost in the absence of *amgR* (Fig.3D; see strain K3615). Thus, AG-inducible *mexXY* expression is not a simple matter of ArmZ-mediated MexZ derepression but also requires activation or operation of the AmgRS TCS.

### Involvement of AmgRS-regulated protease genes in AG-inducible *mexXY* expression

**Figure 5:**
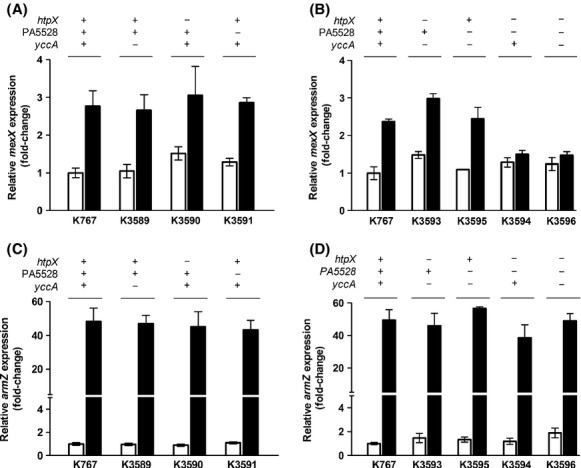
Contribution of AmgRS target genes to aminoglycoside-inducible *mexXY* expression. Expression of *mexXY* (A and B) and *armZ* (C and D) was assessed in late-log phase cultures of the indicated strains without (open bars) and with (filled bars) exposure to paromomycin using real-time quantitative PCR. The status of the *htpX*, PA5528 and *yccA* genes (+, wild-type gene present; −, gene deleted) in each strain is indicated. Expression was normalized to *rpoD* and is reported relative to the wild-type *Pseudomonas aeruginosa* strain K767 (fold-change). Values are means ± standard errors of the means (SEMs) from at least three independent determinations, each performed in triplicate.

Nine genes show AmgRS-dependent induction by the AG tobramycin (Lee et al. 2009), of which three, *htpX*, *yccA,* and PA5528, were primarily responsible for this TCS's contribution to the intrinsic AG resistance of *P. aeruginosa* (Hinz et al. 2011). The *htpX* and *yccA* homologues in *E. coli* encode a CM-associated protease (Sakoh et al. 2005) and a modulator of the FtsH protease (van Stelten et al. 2009), respectively, with both proteases implicated in membrane protein quality control (Sakoh et al. 2005; van Stelten et al. 2009). PA5528 encodes a predicted CM-associated protein of unknown function. Since MexXY is recruited in response to AmgRS activation (by mutation or AG exposure) and proteases are central to the AmgRS-regulated response to AGs (Hinz et al. 2011), we reasoned that *mexXY* induction might be dependent on this protease activity, possibly promoted by the degradation products of AG-generated mistranslated polypeptides. These products might, in turn, be the inducers and intended substrates for MexXY. To test this, the aforementioned AmgRS-regulated genes were deleted individually and in combination, and the resultant deletion strains were assessed for AG-inducible *mexXY* expression. Single knockouts in *htpX*, PA5528, or *yccA* had no impact on AG- (i.e., PAR-) inducible expression of *mexXY* (Fig.5A). Mutants lacking *yccA* in combination with *htpX* or PA5528 also retained PAR-inducible *mexXY* expression (Fig.5B). In contrast, PAR-inducible *mexXY* expression was absent in the HtpX^-^ PA5528^-^ double-knockout strain K3594, and the further elimination of *yccA* in the triple knockout stain K3596 had no additional adverse impact on *mexXY* expression (Fig.5B). Thus, *htpX* and PA5528 are the sole AmgRS-regulated genes that are required for AmgRS-dependent PAR-inducible *mexXY* expression. Loss of *htpX*, PA5528, and *yccA*, alone or in combination, had no effect on *armZ* expression (Fig.5C and D), consistent with the AmgRS independence of and, so, lack of a need for AmgRS targets for PAR-inducible *armZ* expression.

## Discussion

Although inducible by ribosome-perturbing agents and mutations, it is clear that *mexXY* “inducers” are not created equally, with SPC, for example, promoting substantial induction of *mexXY* (Jeannot et al. 2005; Hay et al. 2013) (this study) while AGs provide for very modest induction of this efflux system (Jeannot et al. 2005) (this study). This speaks to possibly different pathways to the recruitment of the MexXY-OprM efflux system by ribosome-perturbing agents. Indeed, the current study demonstrates that AGs, unique amongst ribosome-targeting antimicrobials in promoting mistranslation and generating membrane-damaging aberrant polypeptides (Davis et al. 1986; Busse et al. 1992), upregulate the *mexXY* operon by activating the AmgRS TCS that responds to AG-promoted membrane damage and that protects *P. aeruginosa* from this damage, with AG induction of *mexXY* dependent on AmgRS. In common with non-AG inducers of *mexXY*, AG induction of *mexXY* is, however, also dependent on the ArmZ anti-repressor of MexZ, an indication that AmgR activation of this efflux operon still requires loss of MexZ repression of *mexXY*. Consistent with these regulators operating independently of one another, *armZ* expression was not regulated by AmgRS (this study) (loss of *armZ* also had no impact on the expression of the AmgRS-regulated *htpX* and PA5528 genes [data not shown]), and the drug-independent expression of *mexXY* seen in a *mexZ* mutant was not compromised by the absence of *amgR*. In addition, loss of *amgRS* appears only to block AG enhancement of *mexXY* expression, with basal expression levels of *mexXY* retained in AmgRS^−^ strains, while loss of *armZ* has a more significant impact on *mexXY* expression, eliminating AG-inducible expression of *mexXY* and reducing basal level expression of the efflux operon. Presumably, additional endogenous ArmZ-dependent *mexXY* inducers exist in the cell that, as with non-AG antimicrobial inducers of *mexXY*, operate independent of AmgRS (and likely dependent on other regulators).

**Figure 6:**
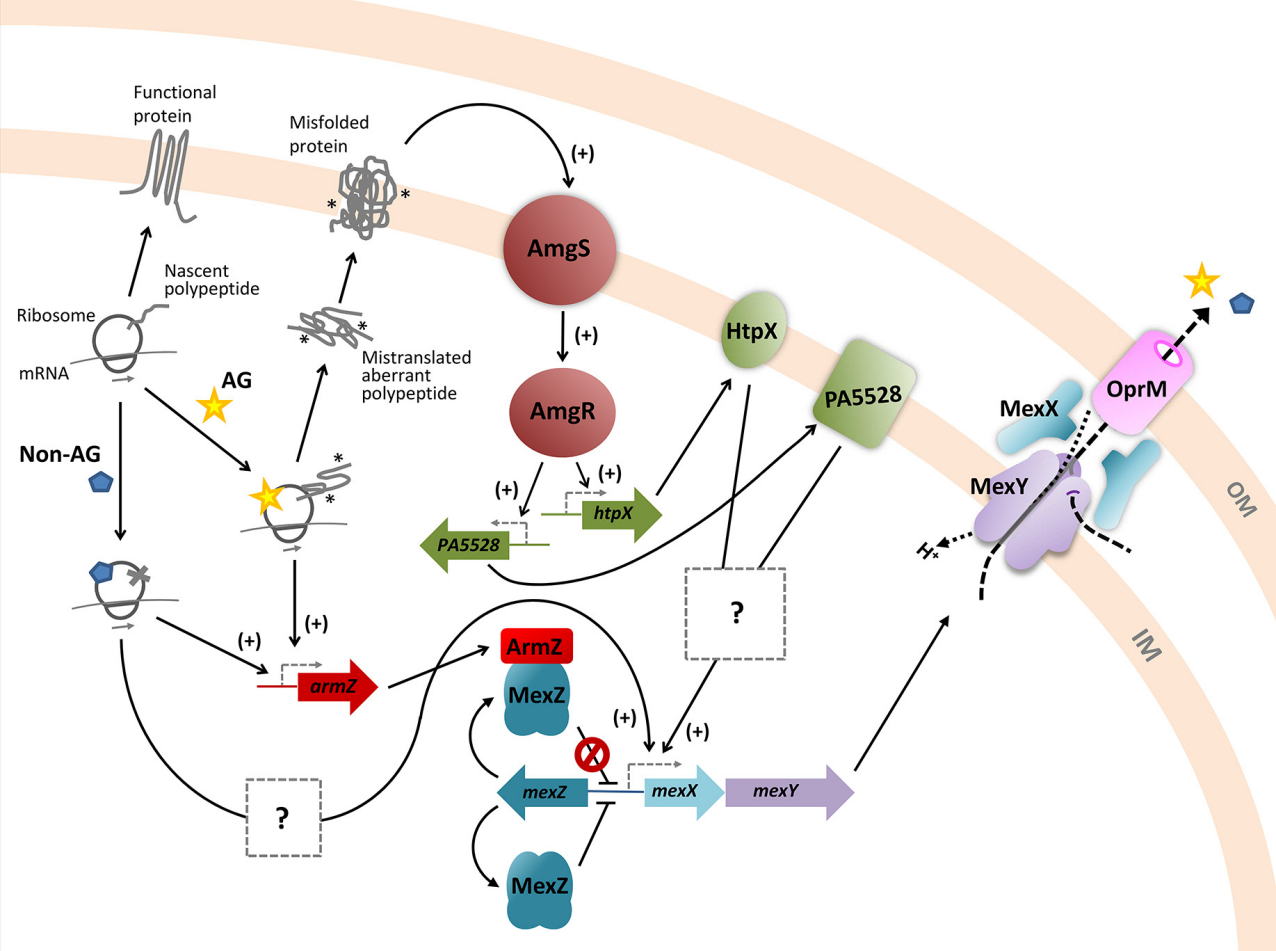
Schematic representation of ArmZ and AmgRS regulation of *mexXY* in *Pseudomonas aeruginosa*. In the absence of ribosome-perturbing agents, protein synthesis occurs normally and native, functional proteins are synthesized. In the presence of ribosome-targeting antimicrobials including aminoglycosides (AG), which promote mistranslation and aberrant polypeptide synthesis, and nonaminoglycosides (non-AG), which halt protein synthesis, expression of the *amrZ* gene is induced. ArmZ, an anti-repressor, modulates the activity of the *mexXY* repressor, MexZ, leading to expression of *mexXY* and, ultimately, production and assembly of the MexXY-OprM multidrug efflux system. Additionally, AG-generated aberrant polypeptides disrupt the inner membrane (IM) activating AmgS, the sensor component of the AmgRS two-component system, which in turn activates AmgR to drive expression of the *htpX* and PA5528 genes. The activities of the *htpX* and PA5528 gene products in some, as yet unknown way promote expression of the *mexXY* operon, dependent on ArmZ-mediated loss of MexZ repression of *mexXY*. Nonaminoglycoside ribosome inhibitors may also promote *mexXY* expression via additional, as yet unknown regulatory pathway(s), also dependent on ArmZ-mediated loss of MexZ repression. OM, outer membrane.

Despite differences in AmgRS dependency amongst *mexXY* inducers it is unclear whether the actual inducing signals and/or efflux substrates that are generated and ultimately accommodated by MexXY-OprM differ in cells treated with AmgRS-dependent (i.e., AGs) versus AmgRS-independent inducers. Since drug-inducible *mexXY* expression is a response to ribosome perturbation and not the drugs themselves (Jeannot et al. 2005) the actual inducing signal(s) are likely generated as a result of some downstream effect(s) of translation disruption. Whether these are common to both classes of inducers and can be generated with or without membrane perturbation is unknown. Still, it is hard to imagine inducers whose generation is dependent on an envelope stress response (AmgRS) and two of its target genes (*htpX,* PA5528) being the same as those generated independent of these. It may be, therefore, that other regulators/regulatory systems mediate *mexXY* induction by non-AGs. The observation, for example, that SPC induction of *mexXY* greatly exceeds that seen in a *mexZ* knockout strain and is retained in a MexZ^−^ *P. aeruginosa* strain (Hay et al. 2013) indicates that MexZ derepression alone does not explain SPC-inducible *mexXY* expression, and suggests that other factor(s)/regulator(s) (but not AmgRS) mediate this. Still, SPC induction of *mexXY* expression also requires loss of MexZ repression, just as AmgRS-dependent AG induction of *mexXY* does, an indication that the downstream-of-ribosome-perturbation signals must similarly be acting through both ArmZ/MexZ as well as the putative additional factor(s)/regulator(s). Thus, the simplest explanation for drug-inducible *mexXY* expression is that some shared feature or downstream effect of ribosome perturbation by AGs and non-AGs promotes MexZ derepression via ArmZ while unique downstream effects of AG versus non-AG perturbation of the ribosome signals though AmgRS (AGs) and other regulators (non-AG ribosome perturbing agents) (Fig.6). Certainly, ribosome-targeting agents have a myriad of effects on bacterial gene expression and physiology (Ng et al. 2003; Sabina et al. 2003; Shaw et al. 2003; Hutter et al. 2004; Aakra et al. 2005; Lin et al. 2005; Qiu et al. 2005; Nanduri et al. 2009), some common and some agent specific (Ng et al. 2003; Sabina et al. 2003; Hutter et al. 2004). Having *mexXY* regulators responsive to drug-specific effects would ensure *mexXY* recruitment in response only to those consequences of translation perturbation where MexXY-OprM function is required. What that function is, and what the intended efflux substrates are, is as yet undetermined, although the observation here that AG-promoted *mexXY* expression is dependent on AmgRS targets that include a presumed protease (HtpX) is consistent with the AG-generated *mexXY* inducer and possible MexXY-OprM substrate being a degradation product(s) of AG-generated aberrant polypeptides. As such, MexXY-OprM may function generally as part of an aberrant polypeptide turnover and detoxification process. Still, if this is the case, then there must be some overlap in HtpX and PA5528 function, at least as regards of generation of the *mexXY* inducer, since loss of either alone has no impact on AG-promoted *mexXY* expression (i.e., not simply a matter of HtpX cleaving mistranslated proteins to yield the *mexXY* inducer). Consistent with HtpX and PA5528 having some shared and, thus, mutually compensatory function, PA5528 expression increases threefold in an *htpX*-mutant strain (data not shown). Although SPC treatment does not yield membrane-damaging aberrant polypeptides, it may well yield truncated/prematurely terminated polypeptides as a result of its interference with the translocation step of translation (Peske et al. 2004). SPC does not appear to fully block translation (Peske et al. 2004), perhaps allowing for partial synthesis of some translation products, with accumulation of these truncated polypeptides possibly having their own deleterious effects on the cell. As such, they may need to be turned over by another (not AmgRS) stress response system and, perhaps, its own set of proteases, with MexXY-OprM again possibly responsible for the export of their degradation products.

The observation that the increased *mexXY* expression of the amgS_V121G_ mutant is ArmZ independent is puzzling, contrasting as it does with the ArmZ dependence of drug- (Morita et al. 2006) and ROS- (Fraud and Poole 2011) inducible *mexXY* expression. Whether this reflects the presence of a truly ArmZ-independent route to *mexXY* recruitment in *P. aeruginosa* is, at present, unknown. It is worth noting, however, that not all *amgS* gain-of-function mutations impact *mexXY* expression – the R182C mutation does not – an indication that AmgRS activation alone is insufficient to drive *mexXY* expression. Presumably, this speaks to some unique feature of the *amgS*_V121G_ mutation and its impact on AmgRS signaling. Equally puzzling is the observation of decreased resistance to non-AG inducers/substrates of *mexXY* in the *amgS*_V121G_ mutant, despite the increase in *mexXY* expression that results from this *amgS* mutation. This suggests that the operation of the MexXY-OprM pump is being compromised in some way in the *amgS*_V121G_ mutant. The failure of the *amgS*_V121G_ mutation to adversely impact resistance to AGs, which are also *mexXY* inducers/MexXY-OprM substrates, is presumably due to the positive impact of AmgRS activation on non-MexXY contributors to AG resistance. Consistent with this, the *amgS*_V121G_ mutation promoted AG resistance in the absence of *mexXY*. In addition, the observation that the positive impact of the *amgS*_V121G_ mutation on resistance to some AGs was actually greater in the absence versus the presence of the pump is in agreement with the MexXY-mediated export of/resistance to AGs also being compromised in the *amgS*_V121G_ mutant (i.e., net impact of the mutation was less in the latter instance owing to the reduced MexXY-OprM contribution to AG resistance). Whatever the explanations for these unique features of the *amgS*_V121G_ mutant, the mutant provided the crucial link between AmgRS and *mexXY*, ultimately confirming a role for the TCS in AG-inducible expression of the efflux system.
